# Supplementary material for: Protein Signatures and Individual Circulating Proteins, including IL-6 and IL-15, Associated with Prognosis in Patients with Biliary Tract Cancer
Source: Cancers (Basel). 2023 Feb 7;15(4):1062. doi: 10.3390/cancers15041062 (PMC9953893; doi:10.3390/cancers15041062)
Supplement: Supplementary file 1 [file cancers-15-01062-s001.zip › 20230204 Supplementary File S1 - suppl. method and figures - Prognostic proteins for BTC - proofread.pdf]

Supplementary Data—Protein Signatures and Individual Circulating Proteins, including IL-6 and IL-15, Associated with Prognosis in Patients with Biliary Tract Cancer

Supplementary methods

Patients with biliary tract cancer from Herlev Hospital

Patients from Herlev hospital were included in one of the following studies:

CHOCA study – "Identification of new biomarkers for patients with cholangiocarcinoma and gallbladder cancer (CHOCA)" is an ongoing prospective observational open-cohort biomarker study of patients with biliary tract cancer (BTC) treated at the Department of Oncology, Herlev Hospital (Clinical.trials.gov.ID: NCT05184400, Regional Ethics Committee approval number: H-3-2014-055, the Danish Data Protection Agency approval number: BBH-2014-097, I-suite nr. 03260, P-2020-797). All patients referred for treatment for BTC were eligible, and patients were included at initiation of adjuvant, neoadjuvant, or palliative treatment (first- and later-treatment lines). They were treated based on the treating physician's choice of different types of chemotherapy, or protocolized treatment with targeted monoclonal antibodies, immunotherapy, and/or radiation. For the present study, only patients who received standard treatment in subsequent treatment line were included. The study was initiated in January 2015. By May 20, 2021, 257 patients had been included in the CHOCA study. Of these, 86 were eligible for inclusion and had baseline serum samples collected before first-line treatment (n = 81), second-line (n = 3), or before both first- and second-line (n = 2).

Clinical trial GI1003 – "Intra-hepatic chemotherapy with oxaliplatin every second week in combination with systemic gemcitabine and capecitabine and in patients with a KRAS-wild type tumor in combination with cetuximab in patients with non-resectable liver metastases from cholangiocarcinoma" [1] (EudraCT: 2010-020188-19, Regional Ethics Committee approval number: H-3-2010-053, the Danish Data Protection Agency approval number HEH.750.24-39). The single center trial included patients with both cholangiocarcinoma and gallbladder cancer, and the inclusion period was from January 2011 to August 2013. In total, 56 patients were included. Of these, 46 were eligible for the

present study and had available baseline serum samples before first-line treatment (n = 43) or second-line treatment (n = 3).

Clinical trial GI1333 – "Randomized first-line treatment with gemcitabine, capecitabine, oxaliplatin vs gemcitabine and cisplatin to patients with cholangiocarcinoma" [2] (EudraCT: 2013-004854-46, Regional Ethics Committee approval number: H-2-2014-026, the Danish Data Protection Agency approval number: I-Suite 02756BBH). The trial included patients with both cholangiocarcinoma and gallbladder cancer. The trial included a total of 100 patients at two sites (Herlev and Vejle Hospitals) from July 2014 to November 2017. In total, 82 patients with locally advanced or metastatic BTC were included in the trial at Herlev Hospital. Of these, 71 were eligible and had available baseline serum samples collected before first-line treatment (n = 67), second-line (n = 2), or before both first- and second-line treatment (n = 2).

Clinical trial GI1312 – "Efficacy and safety of capecitabine, irinotecan, gemcitabine, and bevacizumab as second-line treatment in advanced biliary tract cancer: a phase II study" [3] (EudraCT: 2013-001559-11, Regional Ethics Committee approval number: H-2-2013-079, the Danish Data Protection Agency approval number: HEH-2013-062, I-Suite 02482). The trial included patients with both cholangiocarcinoma and gallbladder cancer. In total 50 patients were included from November 2013 to January 2016. Of these, 44 were eligible for the present study and had available baseline serum samples collected before second-line treatment.

The three trials CHOCA, GI1333, and GI1312 had overlapping inclusion periods. Patients were included in GI1333 as first priority if eligible and consented to inclusion. If they did not participate in GI1333, they were invited to participate in CHOCA. Patients included in both CHOCA study and GI1333 before first-line treatment were allowed to be included in GI1312 for second-line treatment.

Patients with biliary tract cancer from Rigshospitalet

Patients from Rigshospitalet had all been included in the BIOPAC study "Biomarkers in patients with pancreatic cancer", an ongoing prospective open-cohort multi-center biomarker study of patients referred for treatment for pancreatic cancer to one of six hospitals in Denmark (Clinical.trials.gov.ID: NCT03311776, Regional Ethics Committee approval number: KA-20060113, the Danish Data Protection Agency approval number: 2012-58-0004, HGH-2015-027, I-Suite 03960, PACTICUS P-

2020-834). A subset of patients was included on suspicion of pancreatic cancer prior to surgical resection or diagnostic biopsy at the Department of Surgery, Rigshospitalet, Copenhagen, Denmark, and were later diagnosed with BTC. Patients had blood samples collected before the surgical procedure. Patient with resectable disease had tumor resected at the Department of Surgery, Rigshospitalet. Patient with locally advanced or metastatic disease with adequate performance status and limited comorbidities were referred for first-line treatment at the Department of Oncology, Herlev Hospital. As of June 1, 2021, 61 patients with BTC were included in the BIOPAC study prior to a surgical procedure, 48 of whom had baseline serum samples collected before surgery (n = 41) or first-line therapy (n = 7) for BTC and were eligible for inclusion in this study.

Patients with biliary tract cancer from Vejle Hospital

Patients from Vejle hospital had all been included in one of two clinical trials:

Clinical trial GOC-BP - "Randomized phase II trial of combination chemotherapy with panitumumab or bevacizumab for patients with inoperable biliary tract cancer without KRAS exon 2 mutations" [4] (Eudract nr. 2010-020385-13, Regional Ethics Committee approval number: S-20100051, the Danish Data Protection Agency approval number: 2008-58-O035). Eighty-eight patients with locally advanced or metastatic BTC were randomized to receive chemotherapy (gemcitabine, oxaliplatin, and capecitabine) in combination with either panitumumab or bevacizumab from August 2010 to March 2016. Of these, 62 were eligible for the present study and had available baseline plasma samples collected before first-line therapy. As part of the trial, only EDTA plasma and buffy coat were collected.

Clinical trial GOX-P - "Combined biological treatment and chemotherapy for patients with unresectable cholangiocarcinoma" [5, 6] (Eudract nr. 2008-002367-14, Regional Ethics Committee approval number: S-20080081, the Danish Data Protection Agency approval number: 2008-58-O035, 16/1586). The trial was a phase II study where patients were assigned to treatment based on KRAS 2 mutation status. All patients received combination chemotherapy (gemcitabine, oxaliplatin, and capecitabine). Furthermore, patients with KRAS wild-type tumors also received panitumumab. Patients were included from two sites (Vejle, Denmark, Växjö, Sweden). In total, 71 patients were included from October 2008 to 2015, 25 were KRAS exon 2 mutated and 46 were KRAS wild type. Of these, 56 were eligible for the present study and had available baseline plasma samples collected before first-line therapy. As part of the trial, only EDTA plasma and buffy coat were collected.

Olink immune-oncology panel

Blood samples were analyzed using the proximity extension assay (PEA) Olink Target 96 Immuno-Oncology (Olink Proteomics, Sweden). Protein levels were measured on a relative scale and presented as normalized protein expression (NPX), which is an arbitrary unit on log2 scale. A high NPX value corresponds to a high protein concentration [7]. The panel covers proteins previously associated with cancer and the immune system. Several proteins are also linked to systemic inflammation. A full list of proteins is available in Supplementary Table 1.

The analyses were performed at BioXpedia, Aarhus, Denmark. BioXpedia was blinded to the study endpoint as no research questions or clinical data were passed on before all samples had been analyzed. For the analyses, serum and EDTA plasma samples were thawed, mixed using a vortex mixer, and centrifuged at 400 g for 1 minute. Then 1 µL of serum or EDTA plasma was transferred to the incubation plate and mixed with the Olink incubation mix, including antibody-probe pairs, and analyzed according to the manufacturer's instructions. As recommended by Olink, samples were randomized across assay plates. To each plate eight external controls were added: two sample controls (pooled plasma samples), two negative controls, and two inter-plate control (synthetic samples). Likewise, internal controls were added to each sample including incubation control (non-human antigen), extension control, and detection controls [8]. Additionally, we included eight bridging samples on all plates.

Samples were normalized for any plate effects according to manufacturer's recommendations. For all samples quality control was performed, and samples were removed if the standard deviation of the detection and incubation controls on each plate was above 0.2 NPX or the deviation from the median value for each individual sample was above 0.7 NPX. Samples or proteins with more than 10% missing values were removed. Likewise, proteins with more than 90% of the value below the limit of detection were removed. Normalization and quality control was performed by a trained bioinformatician (EM). Blood samples were stored for Effect of storage was tested for all proteins

Routine blood biomarkers

For patients included at Herlev Hospital and Rigshospitalet with missing albumin, CRP, and CA 19-9 results (should have been collected routinely) serum samples were thawed and analyzed for albumin (n = 152), CRP (n = 125), and CA 19-9 (n = 17) at Herlev Hospital in January 2021 and August 2021. CA19-9 was measured using Atellica IM CA19-9 (Siemens Healthineers, Erlangen, Germany), a two-

step sandwich type chemiluminescent immunometric assay. CRP was measured using a sensitive CRP ultra ready-to-use, liquid assay reagent by an immunoturbidimetric method on a fully automated chemistry analyzer (Kit-test SENTINEL CRP Ultra (UD), 11508 UD-2.0/02 2015/09/23). Albumin was analyzed using Atellica CH 930 (Siemens Healthineers, Erlangen, Germany).

After analyzing these samples, data regarding level of CA19-9 were available from 198 patients in the discovery cohort (100%), 102 patients in the first-line validation cohort (87%), 40 patients in the surgery cohort (100%), and 47 patients in the second-line cohort (87%). In the discovery cohort, data on the following were also available from most patients: CRP (186 patients, 94%), neutrophil count (185 patients, 93%), platelet count (198 patients, 100%), lymphocyte count (139 patients, 70%), albumin (162 patients, 82%), and bilirubin (195 patients, 99%).

Generation of protein signatures

Samples with less than 10% missing values were imputed using a k-nearest neighbor imputation algorithm (the function impute.knn from the R-package impute [9]). Before imputation, the abundance levels of the proteins were scaled to unit variance and centered to have a mean equal to zero.

Three sets of protein signatures (including I-O proteins and CA19-9) were generated to discriminate between patients with short and long survival (set 1: OS ≤ 12 months vs. OS > 12 months; set 2: OS ≤ 6 months vs. OS > 18 months; and set 3: OS ≤ 3 months vs. OS > 24 months). To generate each set of protein signatures the discovery cohort was split randomly into a detection set (1/2 of the data) and a replication set (1/2 of the data). A 500-fold logistic LASSO regression (the R-package glmnet [10]) was used to identify the proteins most strongly associated with survival. For each protein, it was noted how many of the 500 logistic LASSO regression models included that protein as a predictor, and a proportion score was calculated. The proportion score was used as a measurement of the stability of the protein as a predictor of survival. Next signatures were generated based on the proportion score using incremental steps of 5% between each signature, thereby generating up to 21 signatures. Duplicated signatures were removed. Signatures were fitted on the detection set using Ridge Regression (the R-package glmnet [10]).

Comparing biomarker level between patients with BTC and controls

We have previously tested the diagnostic use of 80 of the 89 analyzed proteins in the present study (manuscript submitted). However, the following 8 proteins were not tested in the earlier study:

lymphocyte activation gene 3 protein (LAG3), IL-15, mucin-16 (MUC-16), killer cell immunoglobulin-like receptor 3DL1 (KIR3DL1), tumor necrosis factor (TNF), interferon-gamma (IFN-gamma), IL-33, and arginase-1 (ARG1). To test the ability to discriminate patients with BTC from non-cancer patients, we compared the biomarker level in patients with BTC with the level in 49 patients who had an endoscopic retrograde cholangiopancreatography (ERCP) performed due to a benign biliary tract disease [11]. The reasons for ERCP were image confirmed choledocholithiasis (n = 25), elevated liver enzymes/jaundice (n = 20), and cholangitis (n = 4). Samples were collected during follow-up. Results available in Figure S6.

Supplementary figures

Figure S1. Storage time and protein level.

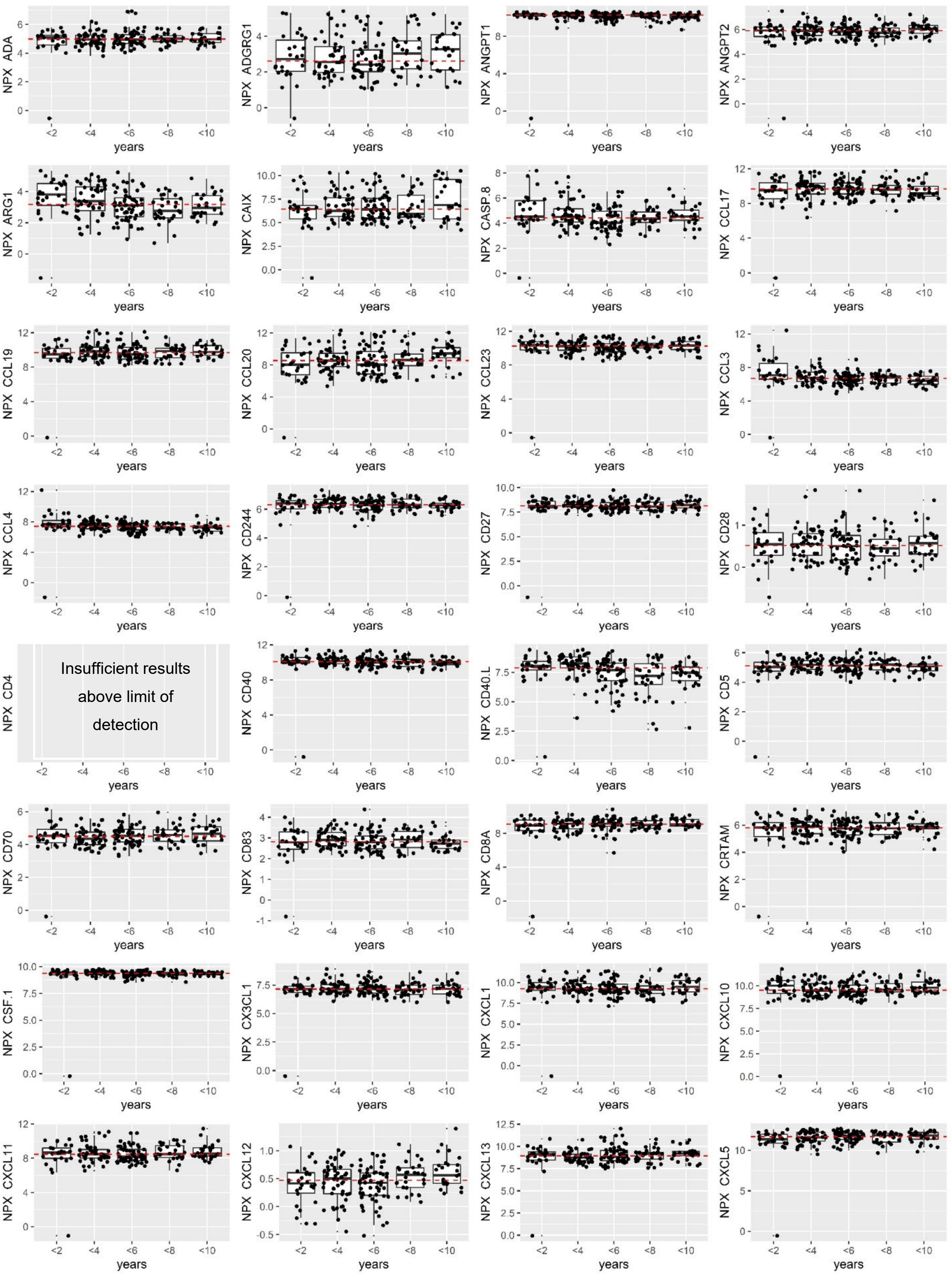

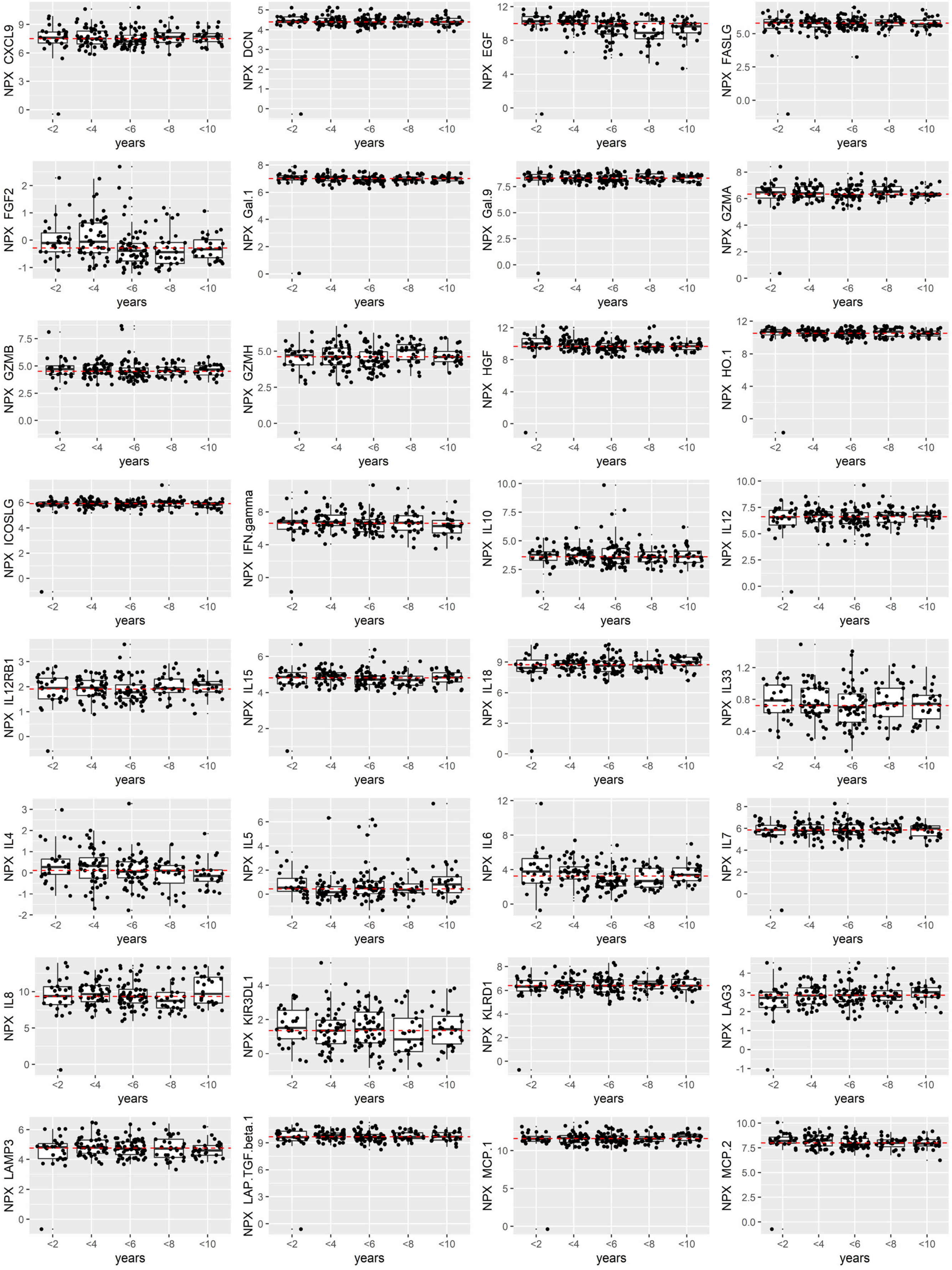

NPX CXCL9
NPX DCN
NPX EGF
NPX FASLG
NPX FGF2
NPX Gal.1
NPX Gal.9
NPX GZMA
NPX GZMB
NPX GZMH
NPX HGF
NPX HO.1
NPX ICOSLG
NPX IFN.gamma
NPX IL10
NPX IL12
NPX IL12RB1
NPX IL15
NPX IL18
NPX IL33
NPX IL4
NPX IL5
NPX IL6
NPX IL7
NPX IL8
NPX KIR3DL1
NPX KLRD1
NPX LAG3
NPX LAMP3
NPX LAP.TGF.beta.1
NPX MCP.1
NPX MCP.2
<2
<4
<6
<8
<10
years

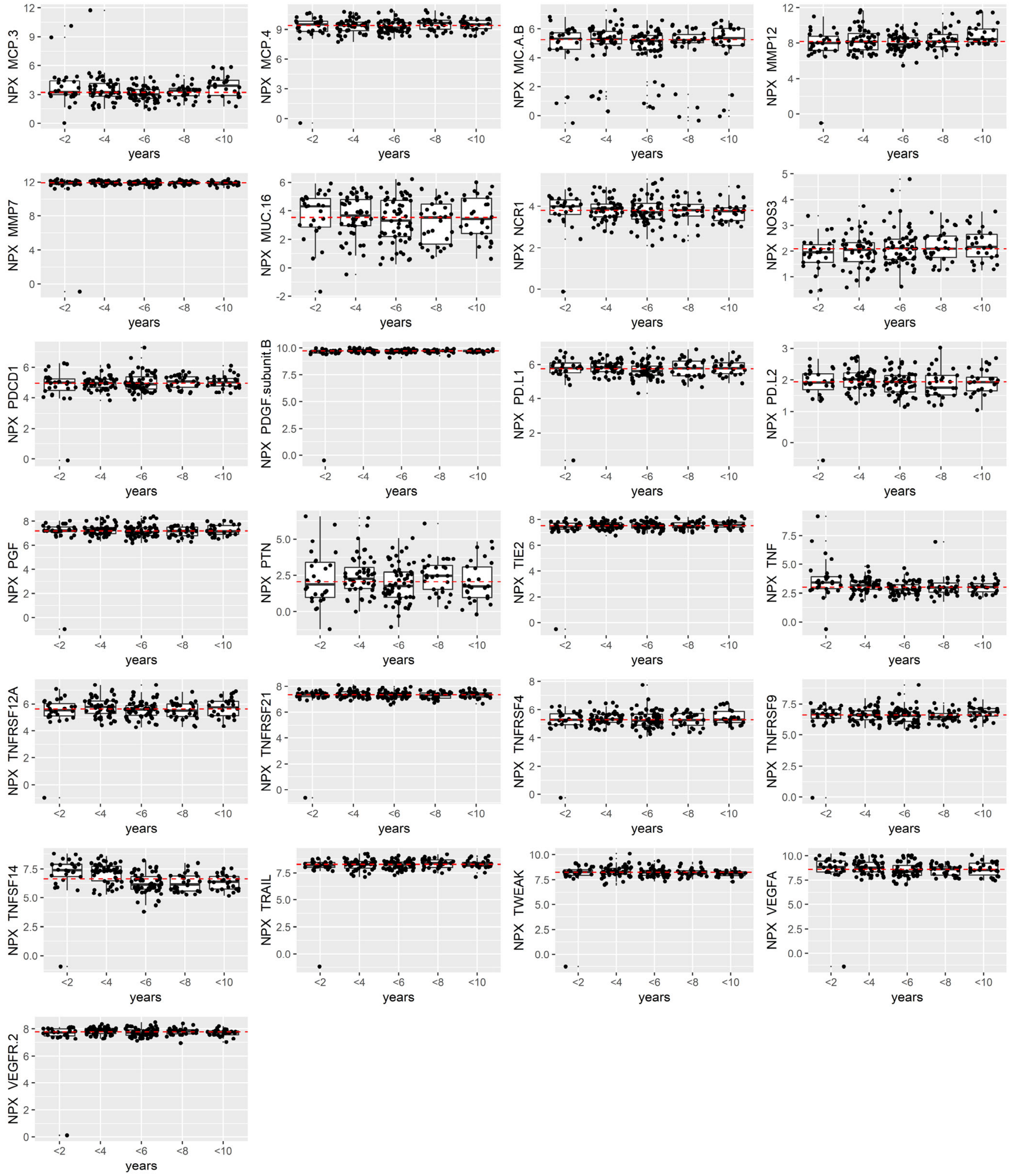

Protein level for all proteins and serum samples included in the discovery cohort according to years from sample collected to date of analyses. All samples were stored at -80°C between collection and analyses. Red dashed line indicates median protein level in the discovery cohort.

Figure S2. Kaplan-Meier plot of survival according to biomarker level in first-line validation cohort.

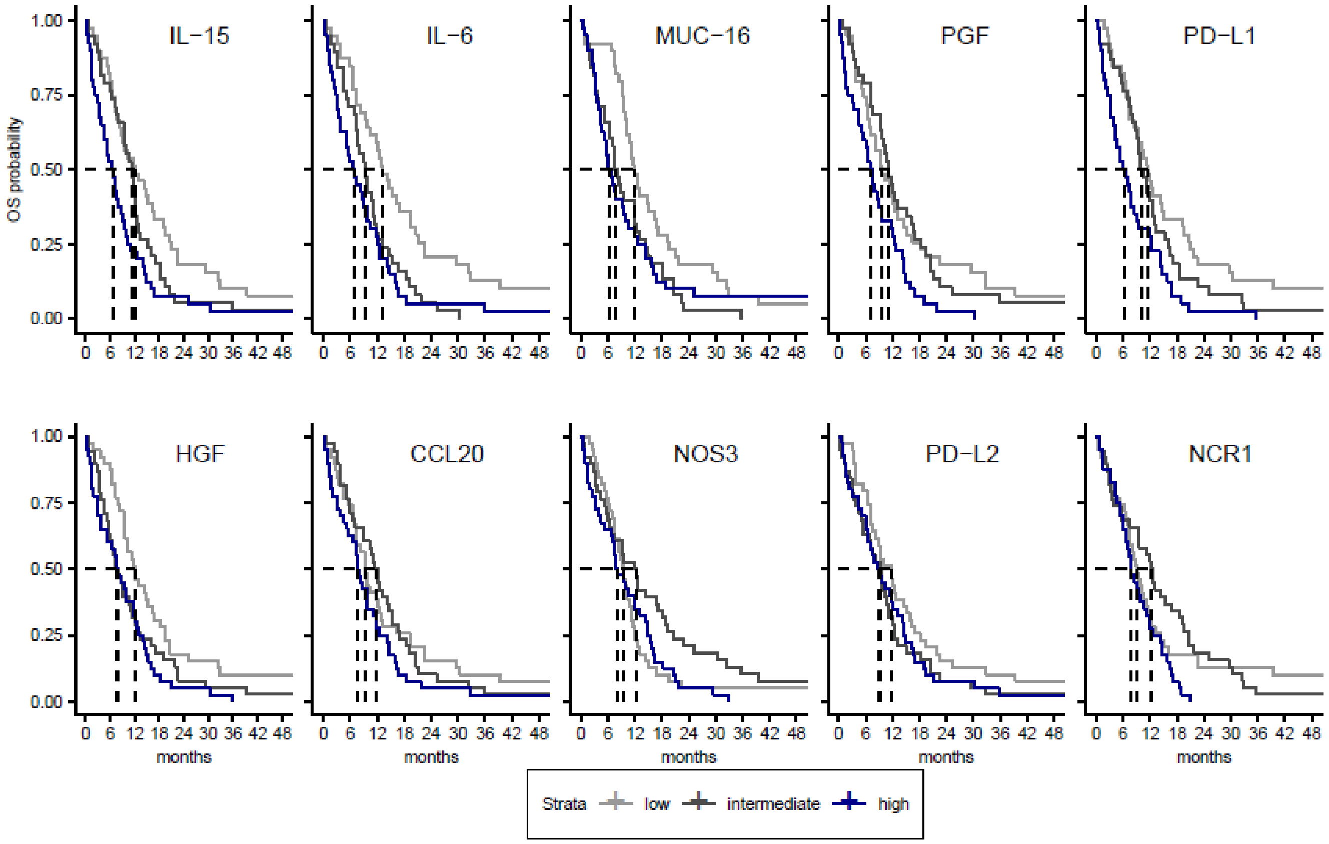

Biomarker level divided into tertiles (low, intermediate, or high).

Abbreviations: CCL20, C-C motif chemokine 20; HGF, hepatocyte growth factor; IL, interleukin; NCR1, natural cytotoxicity triggering receptor; NOS3, endothelial nitric oxide synthase; MUC-16, mucin 16; PD-L1, programmed cell death 1 ligand 1; PD-L2, programmed cell death 1 ligand 2; PGF, placental growth factor.

Figure S3. Protein levels in patients with short and long survival in the discovery cohort.

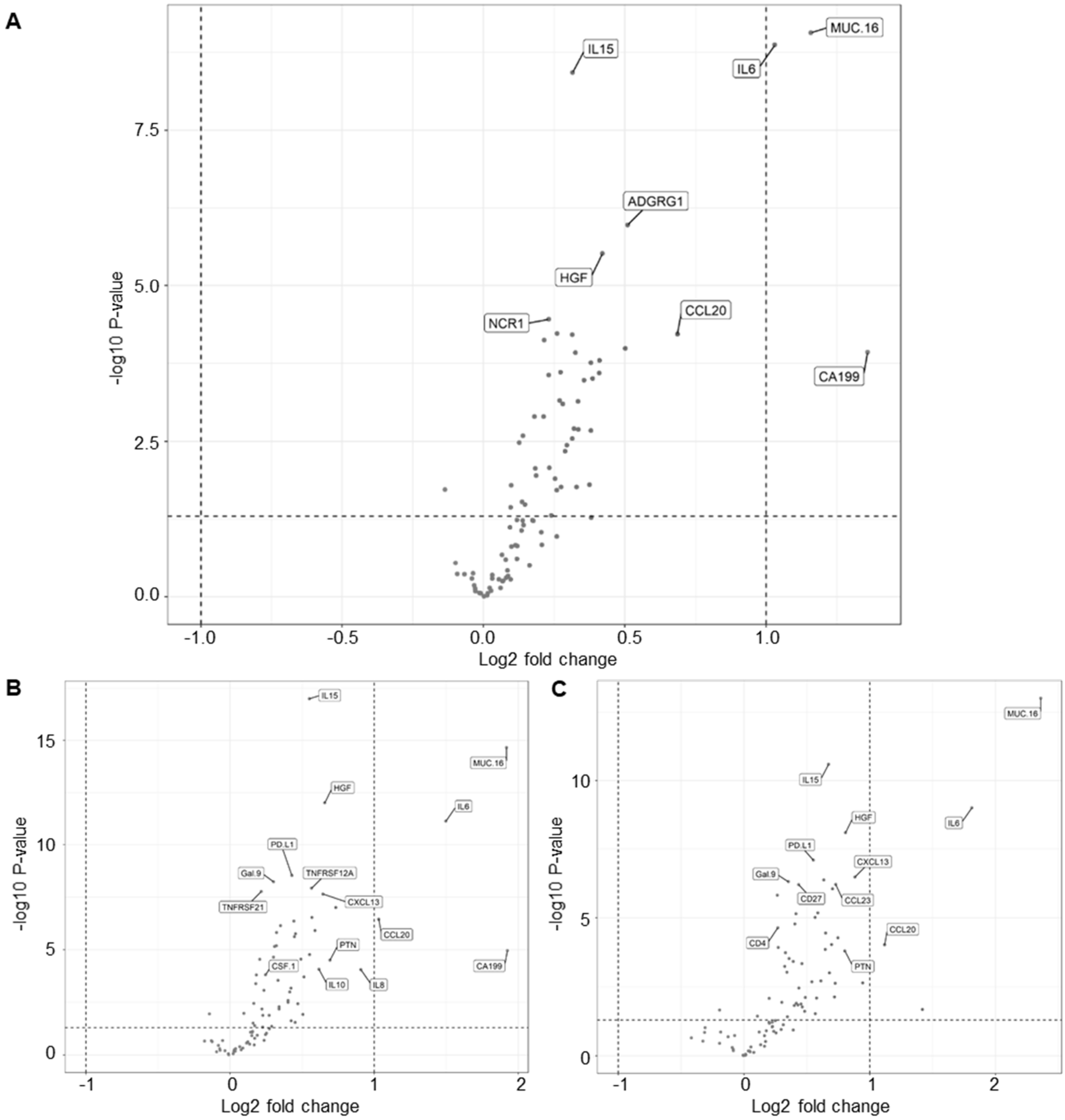

Volcano plots showing difference (log2 fold change) in protein level between patients with short and long survival for all 90 proteins and CA19-9. Positive log2 fold change indicates increased level in patients with short survival. Vertical dashed lines indicate a log2 fold change of 1. Horizontal dashed lines indicate a *p*-value of more than 0.05. The following comparisons are shown: (A) Patients with overall survival (OS) ≤ 12 months vs. OS > 12 months. (B) Patients with OS ≤ 6 months vs. OS > 18 months. (C) Patients with OS ≤ 3 months vs. OS > 18 months.

Abbreviations: ADGRG1, adhesion G-protein coupled receptor G1; CCL, C-C motif chemokine; CD, cluster of differentiation; CSF-1, macrophage colony-stimulating factor 1; CXCL, C-X-C motif chemokine; CA19-9, carbohydrate antigen 19-9; Gal-9, halectin-9; HGF, hepatocyte growth factor; IL, interleukin; NCR1, natural cytotoxicity triggering receptor; NOS3, endothelial nitric oxide synthase; MUC-16, mucin 16; PD-L1, programmed cell death 1 ligand 1; PD-L2, programmed cell death 1 ligand 2; PGF, placental growth factor; PTN, pleiotrophin; TNFRSF, tumor necrosis factor receptor superfamily member.

Figure S4. Correlation between prognostic markers.

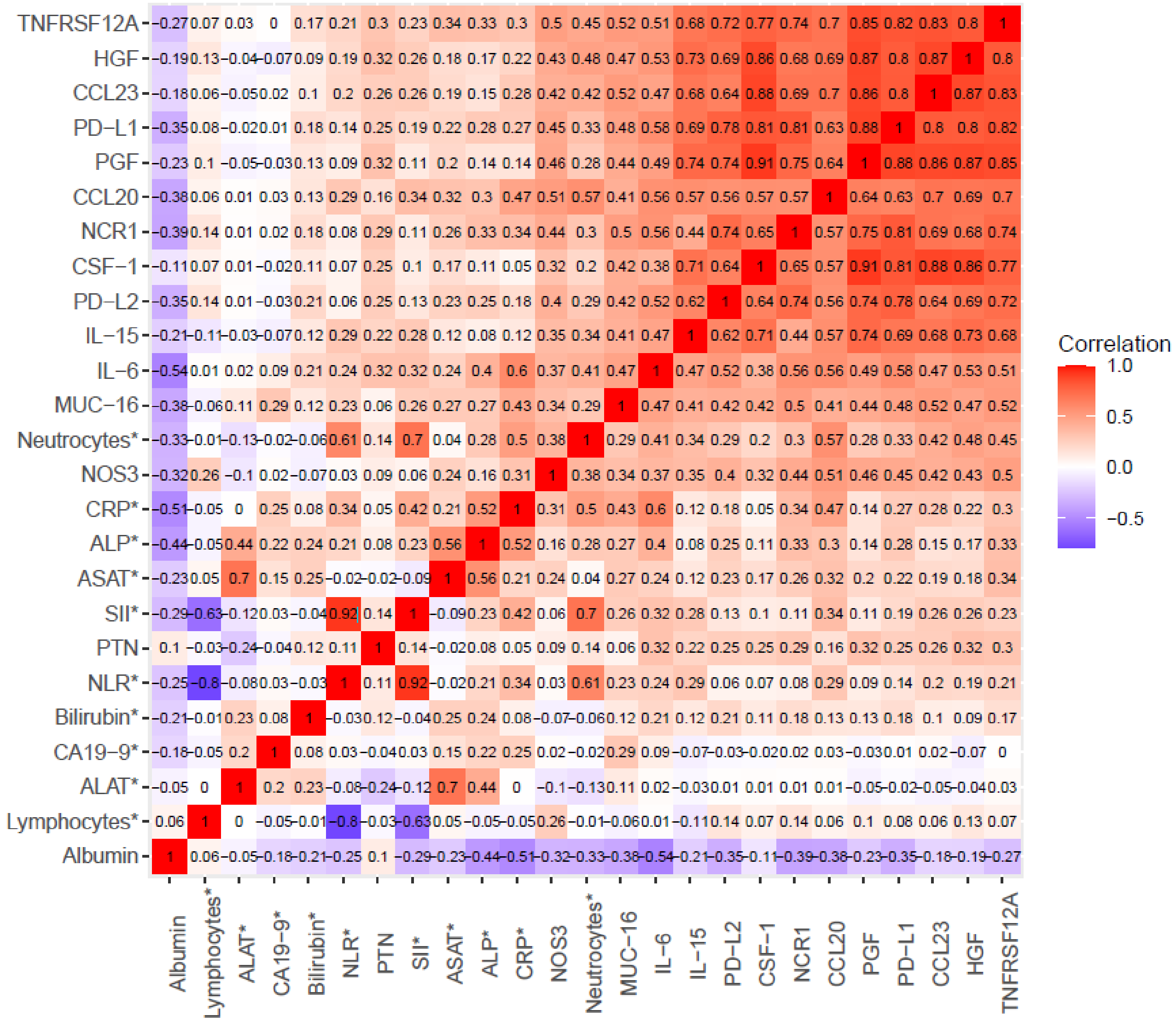

Heatmap showing Pearson correlation between top performing immune-oncology proteins and prognostic biomarkers for overall survival in patients with biliary tract cancer.

*marker transformed using logarithm of 2 to achieve normal distribution.

Abbreviations: ALAT, Alanine transaminase; ALP, alkaline phosphatase; ASAT; Aspartate transaminase; CA19-9, carbohydrate antigen 19-9; CRP, c-reactive protein; HGF, hepatocyte growth factor; IL, interleukin; NLR, neutrophils to lymphocyte ratio (neutrophils/lymphocytes); MUC-16, mucin 16; PD-L1, programmed cell death 1 ligand 1; PD-L2, programmed cell death 1 ligand 2; PGF, placenta growth factor; SII, systemic inflammation index (platelets x neutrocytes/lymphocytes).

Figure S5. Level of IL-15 and IL-6 according to cohort and stage

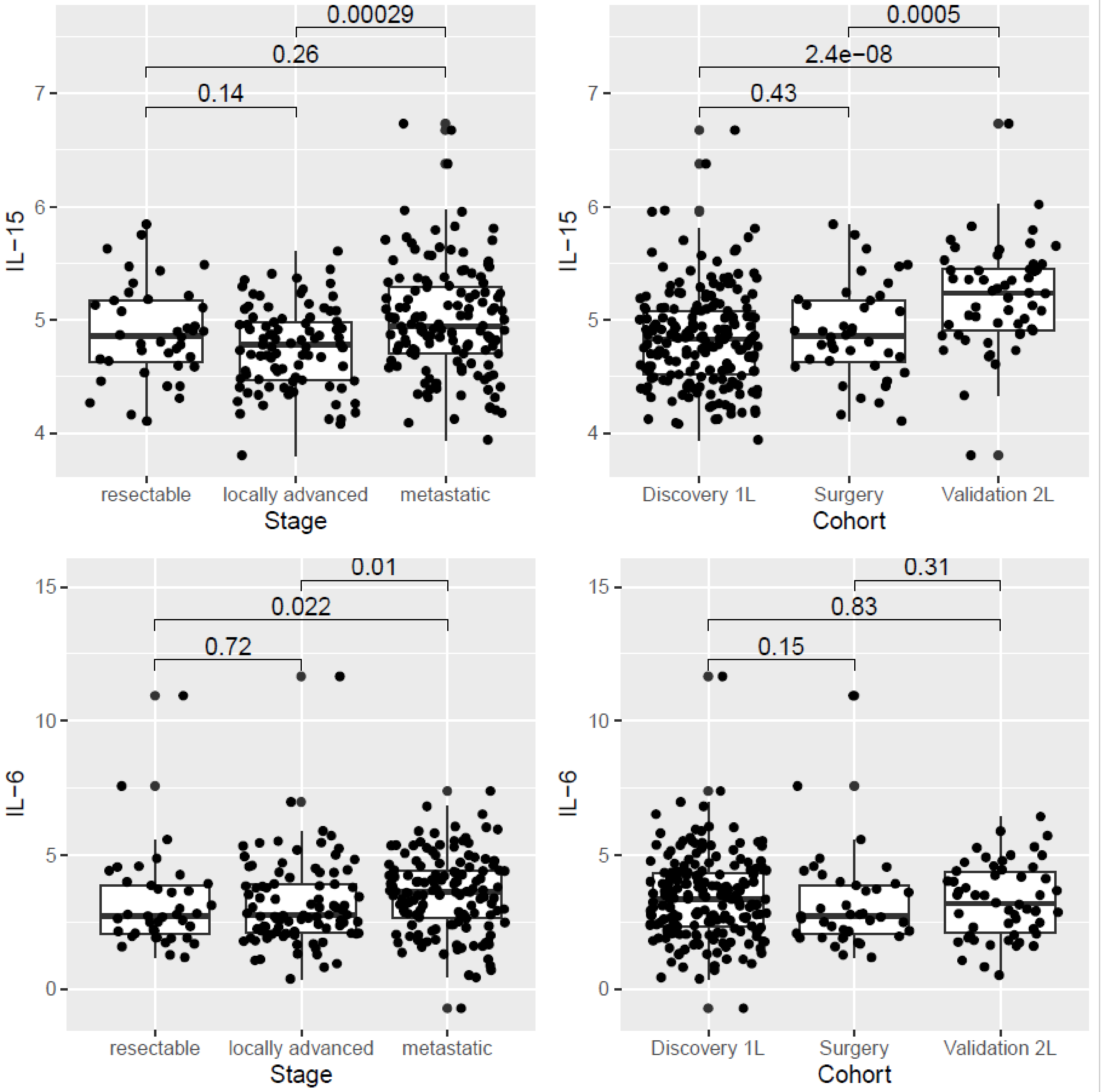

Boxplot showing level of Interleukin (IL)-15 and IL-6 in serum samples according to stage and cohort. Only data from first available serum sample included.

Abbreviation: 1L, first line treatment; 2L, second-line treatment

Figure S6. Proportion scores for all proteins in all models.

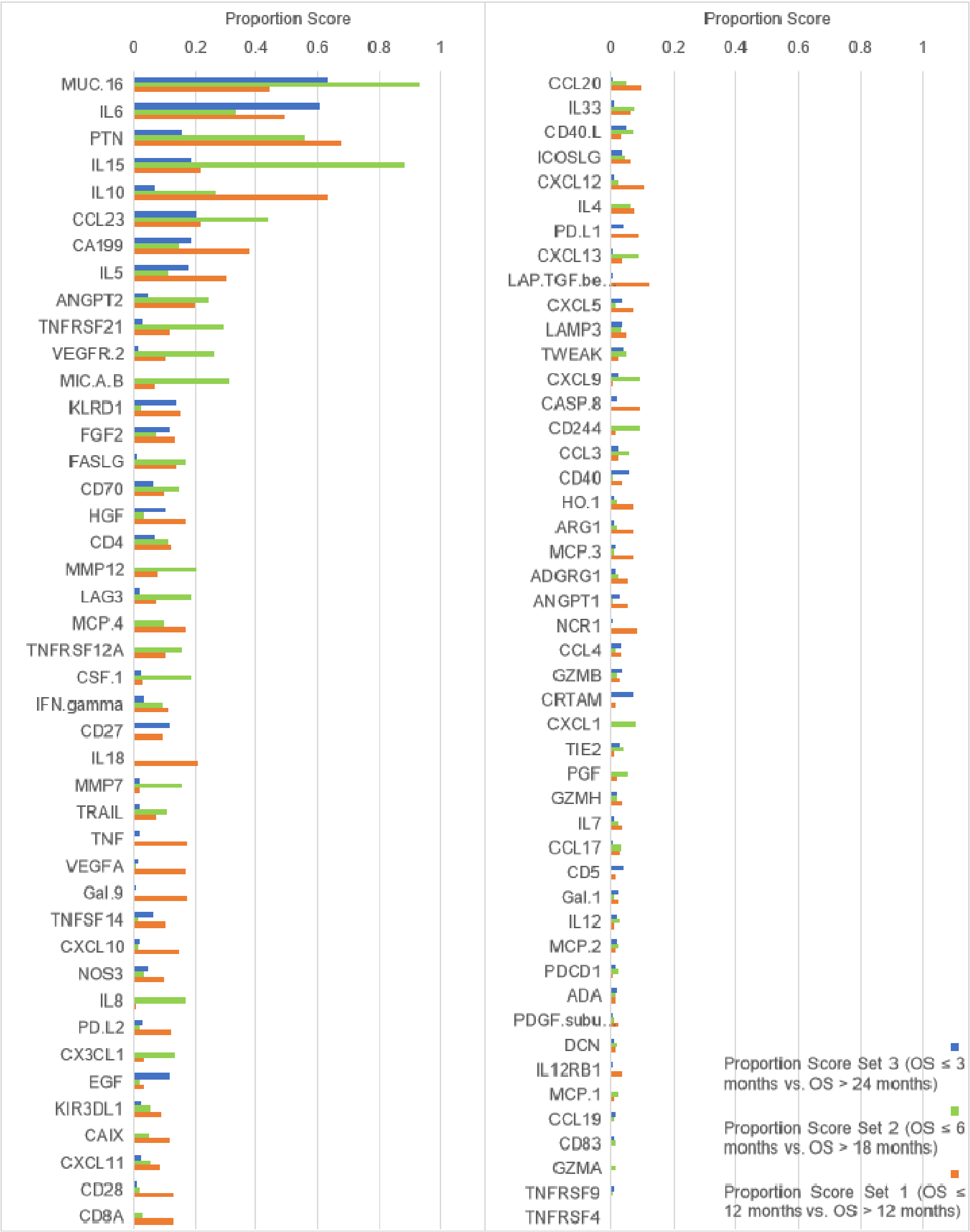

Proportion scores calculated using LASSO regression applied on the detection set of the discovery cohort. Three sets of proportion scores were generated to discriminate between BTC patients with short and long overall survival (OS) (set 1: OS ≤ 12 months vs. OS > 12 months; set 2: OS ≤ 6 months vs. OS > 18 months; and set 3: OS ≤ 3 months vs. OS > 24 months).

Figure S7. Difference in protein level between patients with BTC and controls.

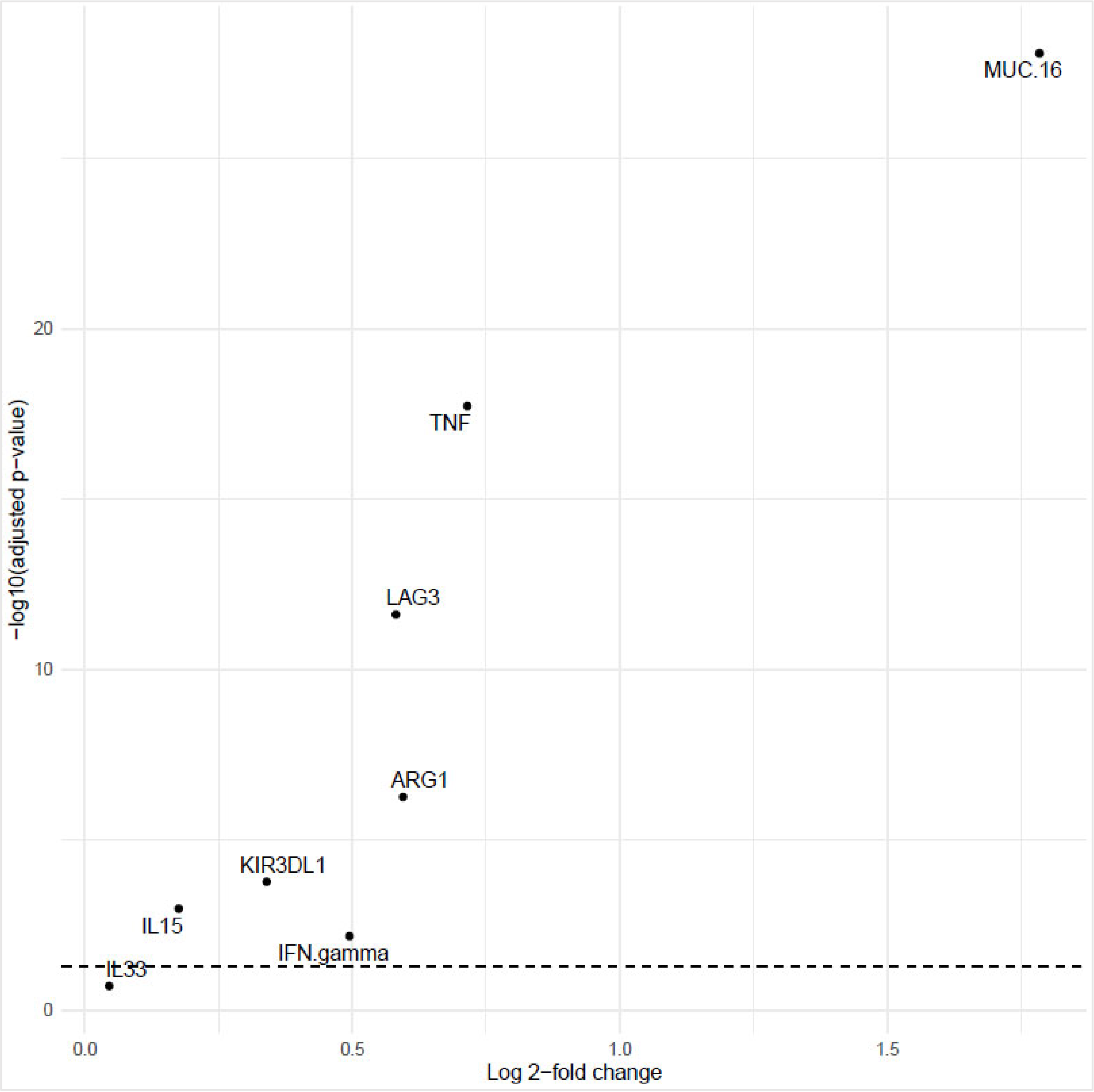

Volcano plot showing log2 fold change and adjusted *p*-value for 8 proteins not tested previously. Dashed line represents an adjusted *p*-value of 0.05. Of the 8 tested proteins, 7 (not IL-33) were significantly elevated in patients with BTC as compared with controls.

Abbreviations: ARG1, arginase-1; IL, interleukin; IFN.gamma, interferon gamma; KIR3DL1, killer cell immunoglobulin-like receptor 3DL1; LAG3, lymphocyte activation gene 3 protein; MUC.16, mucin 16; and TNF, tumor necrosis factor.

References

1. Larsen, F.o., et al., *Gemcitabine, capecitabine and oxaliplatin with or without cetuximab in advanced biliary tract carcinoma.* Acta Oncol, 2016. **55**(3): p. 382-385.

2. Markussen, A., et al., *Treatment of patients with advanced biliary tract cancer with either oxaliplatin, gemcitabine, and capecitabine or cisplatin and gemcitabine-a randomized phase II trial.* Cancers (Basel), 2020. **12**(7): p. 1975.

3. Larsen, F.O., et al., *Efficacy and Safety of Capecitabine, Irinotecan, Gemcitabine, and Bevacizumab as Second-Line Treatment in Advanced Biliary Tract Cancer: A Phase II Study.* Oncology, 2018. **94**(1): p. 19-24.

4. Amin, N.E.L., et al., *Randomized Phase II trial of combination chemotherapy with panitumumab or bevacizumab for patients with inoperable biliary tract cancer without KRAS exon 2 mutations.* Int J Cancer, 2021. **149**(1): p. 119-126.

5. Jensen, L.H., et al., *Phase II marker-driven trial of panitumumab and chemotherapy in KRAS wild-type biliary tract cancer.* Ann Oncol, 2012. **23**(9): p. 2341-2346.

6. Jensen, L., et al., *Phase II study of gemcitabine, oxaliplatin and capecitabine in patients with KRAS exon 2 mutated biliary tract cancers.* Acta Oncol, 2019. **59**: p. 1-4.

7. Assarsson, E., et al., *Homogenous 96-plex PEA immunoassay exhibiting high sensitivity, specificity, and excellent scalability.* PLoS One, 2014. **9**(4): p. e95192.

8. *Olink User Manual v. 1.9.5*. 2018, Olink Proteomics, Uppsala Sweden.

9. Hastie, T., et al., *impute: Imputation for microarray data. R package version 1.58.0.* 2019.

10. Friedman, J., T. Hastie, and R. Tibshirani, *Regularization paths for generalized linear models via coordinate descent.* J Stat Softw, 2010. **33**(1): p. 1-22.

11. Leerhøy, B., et al., *Pancreatic function following post-endoscopic retrograde cholangiopancreatography pancreatitis: A controlled cohort study with long-term follow-up.* United Eur Gastroent J, 2018. **6**(4): p. 586-594.
